# Comparison of Aggregometry with Flow Cytometry for the Assessment of Agonists´-Induced Platelet Reactivity in Patients on Dual Antiplatelet Therapy

**DOI:** 10.1371/journal.pone.0129666

**Published:** 2015-06-09

**Authors:** Thomas Gremmel, Renate Koppensteiner, Simon Panzer

**Affiliations:** 1 Division of Angiology, Department of Internal Medicine II, Medical University of Vienna, Vienna, Austria; 2 Department of Blood Group Serology and Transfusion Medicine, Medical University of Vienna, Vienna, Austria; Baker IDI Heart and Diabetes Institute, AUSTRALIA

## Abstract

Data on the agreement between aggregometry and platelet activation by flow cytometry regarding the measurement of on-treatment platelet reactivity to arachidonic acid (AA) and adenosine diphosphate (ADP) are scarce. We therefore sought to compare three platelet aggregation tests with flow cytometry for the assessment of the response to antiplatelet therapy. Platelet aggregation in response to AA and ADP was determined by light transmission aggregometry (LTA), the VerifyNow assays, and multiple electrode aggregometry (MEA) in 316 patients receiving aspirin and clopidogrel therapy after angioplasty with stent implantation. AA- and ADP-induced P-selectin expression and activated glycoprotein (GP) IIb/IIIa were determined by flow cytometry. LTA, the VerifyNow P2Y_12_ assay and MEA in response to ADP correlated significantly (all p<0.001), and the best correlation was observed between LTA and the VerifyNow P2Y_12_ assay (r = 0.63). ADP-induced platelet reactivity by all aggregation tests correlated significantly with ADP-induced P-selectin expression and activated GPIIb/IIIa (all p<0.001). The best correlation was seen between the VerifyNow P2Y_12_ assay and activated GPIIb/IIIa (r = 0.68). The platelet surface expressions of P-selectin and activated GPIIb/IIIa in response to ADP were significantly higher in patients with high on-treatment residual platelet reactivity (HRPR) to ADP by all test systems (all p<0.001). A rather poor correlation was observed between AA-induced platelet reactivity by LTA and the VerifyNow aspirin assay (r = 0.15, p = 0.007), while both methods did not correlate with MEA. AA-induced platelet reactivity by all aggregation tests correlated significantly, but rather poorly with AA-induced P-selectin expression (all p<0.05), while only AA-induced platelet reactivity by LTA correlated significantly with AA-induced activated GPIIb/IIIa (r = 0.21, p<0.001). The platelet surface expression of P-selectin in response to AA was significantly higher in patients with HRPR by LTA AA and MEA AA (both p<0.02). In contrast, P-selectin expression in response to AA was similar in patients without and with HRPR by the VerifyNow aspirin assay (p = 0.5), and platelet surface activated GPIIb/IIIa in response to AA did not differ significantly between patients without and with HRPR to AA by all test systems (all p>0.1). In conclusion, ADP-induced platelet reactivity by aggregometry translates partly into flow cytometry. In contrast, AA-induced platelet reactivity correlates poorly between different platelet aggregation tests, and between aggregometry and flow cytometry. Overall, both approaches capture different aspects of platelet function and are therefore not interchangeable in the assessment of agonists´-induced platelet reactivity. Clinical outcome data are needed to determine which test systems and settings are associated with different *in vivo* consequences.

## Introduction

Dual antiplatelet therapy with aspirin and clopidogrel is the most frequently prescribed antithrombotic regimen following percutaneous angioplasty with stent implantation, and both agents were shown to effectively reduce future ischemic events in patients with atherosclerotic cardiovascular disease [1–3]. However, many patients still experience adverse ischemic outcomes during dual antiplatelet treatment. This observation has prompted the development of assays, which measure residual platelet reactivity to arachidonic acid (AA) and adenosine diphosphate (ADP), and thereby allow an estimation of the inhibitory response to aspirin and clopidogrel. Roughly, these methods can be divided into two groups: platelet aggregation tests (i.e. aggregometry), which measure the extent of platelet aggregation in response to AA and ADP [4–6], and flow cytometry, which determines the surface expression of platelet activation markers after the addition of AA and ADP. Numerous studies linked high on-treatment residual platelet reactivity (HRPR) by the most frequently used platelet aggregation tests, namely light transmission aggregometry (LTA), the VerifyNow P2Y_12_ and aspirin assays, and multiple electrode platelet aggregometry (MEA), with the occurrence of atherothrombotic events after angioplasty and stenting [7–10]. Likewise, recent studies revealed associations of agonists´- induced platelet activation as assessed by flow cytometry with ischemic outcomes in patients with atherosclerotic cardiovascular disease [11, 12]. However, data on the agreement between both approaches regarding the measurement of on-treatment platelet reactivity to AA and ADP are scarce. Since not all laboratories offer both, aggregometry and flow cytometry, these data would be of great value to correctly interpret the results achieved with either of these two approaches. We therefore sought to compare the three most frequently used platelet aggregation tests with flow cytometry for the assessment of residual platelet reactivity to AA and ADP in a large patient cohort receiving dual antiplatelet therapy after angioplasty with stent implantation.

## Materials and methods

### Study Population

The study population consisted of 316 patients on dual antiplatelet therapy after percutaneous intervention with endovascular stent implantation. All patients received daily aspirin (100mg/d) and clopidogrel therapy (75 mg/d).

Exclusion criteria were a known aspirin or clopidogrel intolerance (allergic reactions, gastrointestinal bleeding), a therapy with vitamin K antagonists (warfarin, phenprocoumon, acenocoumarol), treatment with ticlopidine, dipyridamol or nonsteroidal antiinflammatory drugs, a family or personal history of bleeding disorders, malignant paraproteinemias, myeloproliferative disorders or heparin-induced thrombocytopenia, severe hepatic failure, known qualitative defects in thrombocyte function, a major surgical procedure within one week before enrollment, a platelet count <100.000 or >450.000/μl and a hematocrit <30%.

The study protocol was approved by the Ethics Committee of the Medical University of Vienna in accordance with the Declaration of Helsinki and written informed consent was obtained from all study participants.

### Blood sampling

Blood was drawn by aseptic venipuncture from an antecubital vein using a 21-gauge butterfly needle (0.8 x 19 mm; Greiner Bio-One, Kremsmünster, Austria) one day after the percutaneous intervention. To avoid procedural deviations all blood samples were taken by the same physician applying a light tourniquet, which was immediately released and the samples were mixed adequately by gently inverting the tubes. After the initial 3ml of blood had been discarded to reduce procedurally-induced platelet activation, blood was drawn into 3.8% sodium citrate Vacuette tubes (Greiner Bio-One; 9 parts of whole blood, 1 part of sodium citrate 0.129 M/L) for evaluations by LTA and flow cytometry, into 3.2% sodium citrate Vacuette tubes (Greiner Bio-One; 9 parts of whole blood, 1 part of sodium citrate 0.109 M/L) for the VerifyNow P2Y12 and aspirin assays, and into Vacuette tubes containing lithium heparin (18 IU/ ml) for the determinations by MEA. To avoid investigator-related variations of the results, each of the different tests was performed by just one corresponding operator, who was blinded to the results from the other operators.

### Light transmission aggregometry (LTA)

LTA was performed on the APACT 4S Plus aggregometer (LABiTec, Ahrensburg, Germany) as previously described [11]. Citrate-anticoagulated whole blood was centrifuged at 150 × g for 10 minutes at room temperature to obtain platelet rich plasma (PRP). Platelet poor plasma (PPP) was obtained from the remaining specimen by re-centrifugation at 2000 × g for 10 minutes. Platelet counts were not adjusted as median platelet count was 208 G/L (interquartile range 176–251 G/L). PRP was used for the baseline reading and PPP as a reference corresponding to 100% aggregation. Aggregation was performed using ADP (10μM) or AA (final concentration of 0.5 mg/ml; both from Rolf Greiner BioChemica, Flacht, Germany) and optical density was recorded for 10 minutes as platelets began to aggregate. The maximal aggregation response was registered and used for further analyses.

### VerifyNow aspirin assay

The VerifyNow system (Accumetrics, San Diego, CA, USA) is a turbidimetric based optical detection system, which measures platelet-induced aggregation as an increase in light transmittance in whole blood. The assay device contains reagents based on microbead agglutination technology, in particular a lyophilized preparation of human fibrinogen-coated beads, platelet agonists, preservative and buffer.

Citrate-anticoagulated whole blood was automatically dispensed from the blood collection tube into the assay device by the instrument. ADP or AA was incorporated into the assay channel to induce platelet activation and light transmittance increased as activated platelets bound and aggregated fibrinogen-coated beads. The instrument measured this change in optical signal and reported the results in P2Y_12_ (PRU) or Aspirin Reaction Units (ARU). With this assay, higher PRU and ARU reflect greater ADP- and AA-mediated platelet reactivity, respectively.

### Multiple electrode platelet aggregometry (MEA)

Whole blood impedance aggregometry was performed with the Multiplate analyzer (Verum Diagnostica, Munich, Germany). One Multiplate test cell contains two independent sensor units and one unit consists of 2 silver-coated highly conductive copper wires with a length of 3.2 mm. After dilution (1:2 with 0.9% NaCl solution) of heparin-anticoagulated whole blood and stirring in the test cuvettes for 3 minutes at 37°C, ADP (6.4 μM) or AA (final concentration of 0.5 mM; both from Verum Diagnostica, Munich, Germany) was added and aggregation was continuously recorded for five minutes. The adhesion of activated platelets to the electrodes led to an increase of impedance, which was detected for each sensor unit separately and transformed to aggregation units (AU) that were plotted against time.

### Determination of P-selectin expression and glycoprotein (GP) IIb/IIIa activation

The expression of P-selectin and the binding of the monoclonal antibody PAC-1 to activated glycoprotein IIb/IIIa (GPIIb/IIIa) were determined in citrate-anticoagulated blood, as previously published [11]. In brief, whole blood was diluted in phosphate-buffered saline to obtain 20x10^3^/μL platelets in 20μl, and incubated for 10 minutes without agonists, and after *in vitro* exposure to suboptimal concentrations of ADP (final concentration 1μM; Dynabyte, Munich, Germany) or AA (final concentration 80 μM; Diamed, Cressier, Switzerland), each 10 μL. The platelet population was identified by staining with anti-CD42b (5 μL of clone HIP1, allophycocyanin labelled, final dilution 1:9; Becton Dickinson (BD), San Jose, CA, USA), and the expression of P-selectin and activated GPIIb/IIIa were determined by the binding of the monoclonal antibodies PAC-1-fluorescein (5 μL, final dilution 1:9; BD) and anti-CD62p-phycoerythrin (5 μL of clone CLB-Thromb6, final dilution 1:9; Immunotech, Beckman Coulter, Fullerton, CA, USA), respectively. Isotype-matched control antibodies were used in separate vials for the determination of non-specific binding. After 15 minutes of incubation in the dark, the reaction was stopped by adding 500 μL PBS and samples were acquired immediately on a FACS Calibur flow cytometer (BD) with excitation by an argon laser at 488 nm and a red diode laser at 635 nm at a rate of 200–600 events per second. FITC and PE labelled beads were used to compensate manually for the FITC signal into the PE channel and vice versa. Platelets were gated in a side scatter versus FL4 dot plot. A total of 10.000 events were acquired within this gate. Positive analysis regions for P-selectin and activated GPIIb/IIIa, respectively, were set with appropriate nonspecific controls. The gated events were further analyzed in histograms for FL-1 and FL-2 for PAC-1 and P-selectin, respectively, using the CellQuest Pro software (BD). Agonists’-induced P-selectin and activated GPIIb/IIIa were calculated as fold change of mean fluorescence intensities (MFI) after the addition of ADP or AA according to the following formula:

Agonists´-induced P-selectin or GPIIb/IIIa = P-selectin or GPIIb/IIIa MFI after the addition of ADP or AA/ P-selectin or GPIIb/IIIa MFI without agonist

Standard BD calibrite beads were used for daily calibration of the cytometer.

### Statistical analysis

Statistical analysis was performed using the Statistical Package for Social Sciences (IBM SPSS version 22, Armonk, New York, USA). Median and interquartile range of continuous variables are shown. Categorical variables are given as number (%). Spearman rank correlations were used to test for correlations between the different methods. Two-sided P-values <0.05 were considered statistically significant.

## Results

Clinical, laboratory and procedural characteristics of the study population are given in Table 1.

**Table 1:** Clinical, laboratory and procedural characteristics of the overall study population.

Characteristics	overall (n = 316)
**Demographics**	
Age, years	66 (58–75)
Body mass index, kg/m^2^	26.8 (24.2–29.7)
**Medical history**	
Hypertension	284 (89.9)
Hypercholesterolemia	294 (93)
Diabetes mellitus	102 (32.3)
Active smoking	133 (42.1)
**Laboratory data**	
Hematocrit, %	38.9 (36–42)
White blood cell count, G/L	8.4 (6.9–10.4)
Platelet count, G/L	208 (176–251)
Serum creatinine, mg/dl	1 (0.9–1.2)
C-reactive protein, mg/dl	1 (0.4–1.9)
**Procedure**	
Stent implantation	316 (100)
Number of stents/patient	1 (1–2)
**Medication pre-intervention**	
Clopidogrel	316 (100)
Aspirin	316 (100)
Statins	301 (95.3)
ACE inhibitors/ARB	273 (86.4)
Beta blockers	217 (68.7)
Calcium channel blockers	96 (30.4)
Proton pump inhibitors	166 (52.5)

Continuous data are shown as median (interquartile range). Dichotomous data are shown as n (%).

ACE inhibitors, angiotensin converting enzyme inhibitors; ARB, angiotensin receptor blockers.

**Fig 1:**
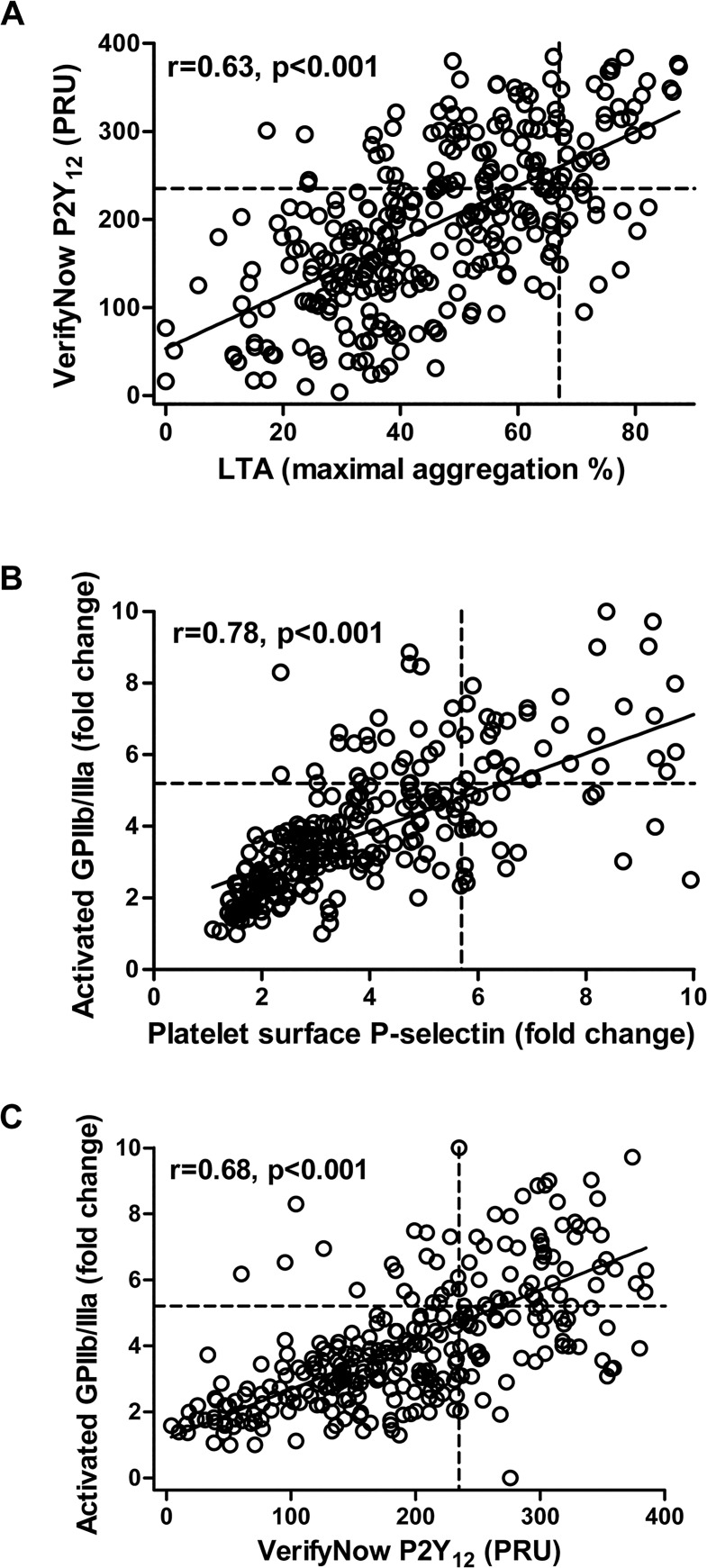
Correlations of adenosine diphosphate (ADP)-induced platelet aggregation and activation. **(A)** Scatter plot showing ADP-induced platelet reactivity by light transmission aggregometry (LTA; x-axis) vs. platelet reactivity by the VerifyNow P2Y_12_ assay (y-axis). Circles represent individual measurements. Cut-off values for high on-treatment residual platelet reactivity are indicated by the dotted lines. PRU, P2Y_12_ Reaction Units. **(B)** Scatter plot showing ADP-induced platelet surface P-selectin expression (x-axis) vs. ADP-induced activated glycoprotein (GP) IIb/IIIa (y-axis). Circles represent individual measurements. Cut-off values for high P-selectin and high GPIIb/IIIa are indicated by the dotted lines. **(C)** Scatter plot showing platelet reactivity by the VerifyNow P2Y_12_ assay (x-axis) vs. ADP-induced activated glycoprotein (GP) IIb/IIIa (y-axis). Circles represent individual measurements. Cut-off values for high on-treatment residual platelet reactivity and high GPIIb/IIIa are indicated by the dotted lines. PRU, P2Y_12_ Reaction Units.

The best correlation regarding ADP-induced platelet reactivity among the platelet aggregation tests was observed between LTA ADP and the VerifyNow P2Y_12_ assay (Fig 1A; r = 0.63, p<0.001). Both tests also correlated significantly with MEA ADP (LTA ADP and MEA ADP: r = 0.46, p<0.001; VerifyNow P2Y_12_ assay and MEA ADP: r = 0.32, p<0.001). Moreover, ADP-induced platelet surface P-selectin expression and activated GPIIb/IIIa correlated strongly (Fig 1B; r = 0.78, p<0.001).

ADP-induced platelet reactivity by all aggregation tests correlated significantly with ADP-induced P-selectin expression and activated GPIIb/IIIa (Table 2). The best correlation was seen between the VerifyNow P2Y_12_ assay and ADP-induced activated GPIIb/IIIa (Fig 1C; r = 0.68, p<0.001).

**Table 2:** Correlations of adenosine diphosphate (ADP)-induced platelet reactivity by light transmission aggregometry (LTA), the VerifyNow P2Y_12_ assay, and multiple electrode aggregometry (MEA) with ADP-induced P-selectin expression and activated glycoprotein (GP) IIb/IIIa.

	P-selectin		Activated GPIIb/IIIa	
Platelet aggregation test	Correlation coefficient	p	Correlation coefficient	P
LTA	0.58	<0.001	0.58	<0.001
VerifyNow P2Y_12_ assay	0.66	<0.001	0.68	<0.001
MEA	0.33	<0.001	0.38	<0.001

**Fig 2:**
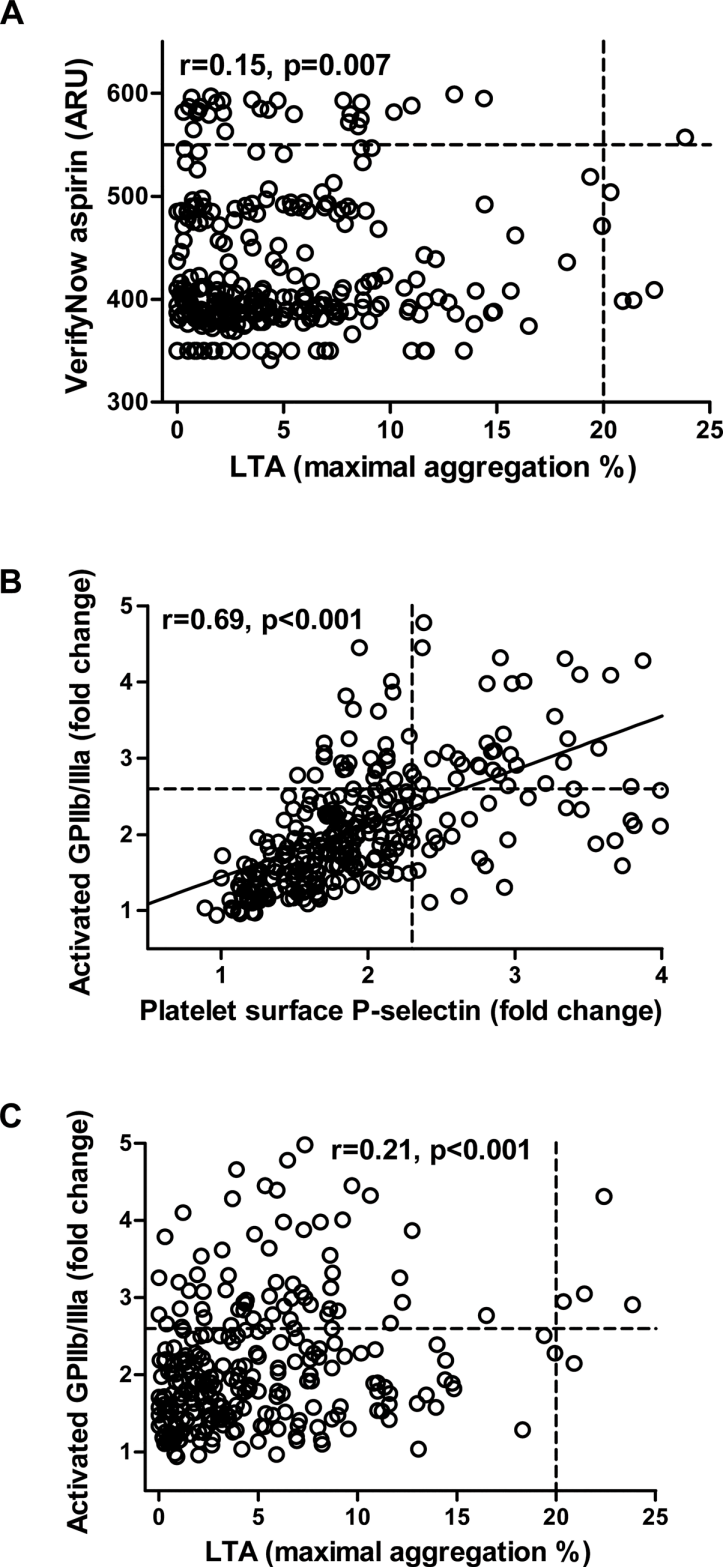
Correlations of arachidonic acid (AA)-induced platelet aggregation and platelet activation. **(A)** Scatter plot showing AA-induced platelet reactivity by light transmission aggregometry (LTA; x-axis) vs. platelet reactivity by the VerifyNow aspirin assay (y-axis). Circles represent individual measurements. Cut-off values for high on-treatment residual platelet reactivity are indicated by the dotted lines. ARU, Aspirin Reaction Units. **(B)** Scatter plot showing AA-induced platelet surface P-selectin expression (x-axis) vs. AA-induced activated glycoprotein (GP) IIb/IIIa (y-axis). Circles represent individual measurements. Cut-off values for high P-selectin and high GPIIb/IIIa are indicated by the dotted lines. **(C)** Scatter plot showing AA-induced platelet reactivity by light transmission aggregometry (LTA; x-axis) vs. AA-induced activated glycoprotein (GP) IIb/IIIa (y-axis). Circles represent individual measurements. Cut-off values for high on-treatment residual platelet reactivity and high GPIIb/IIIa are indicated by the dotted lines. ARU, Aspirin Reaction Units.

The best correlation regarding AA-induced platelet reactivity among the platelet aggregation tests was observed between LTA AA and the VerifyNow aspirin assay (Fig 2A; r = 0.15, p = 0.007). The correlations of both assays with MEA AA did not reach statistical significance (both p≥0.1). AA-induced platelet surface P-selectin expression and activated GPIIb/IIIa correlated strongly (Fig 2B; r = 0.69, p<0.001).

AA-induced platelet reactivity by all aggregation tests correlated significantly, but rather poorly with AA-induced P-selectin expression, while only AA-induced platelet reactivity by LTA correlated significantly with AA-induced activated GPIIb/IIIa (Table 3; Fig 2C).

**Table 3:** Correlations of arachidonic acid (AA)-induced platelet reactivity by light transmission aggregometry (LTA), the VerifyNow aspirin assay, and multiple electrode aggregometry (MEA) with AA-induced P-selectin expression and activated glycoprotein (GP) IIb/IIIa.

	P-selectin		Activated GPIIb/IIIa	
Platelet aggregation test	Correlation coefficient	p	Correlation coefficient	P
LTA	0.12	0.047	0.21	<0.001
VerifyNow aspirin assay	0.18	0.002	0.11	0.06
MEA	0.16	0.007	0.11	0.06

In a second step, we used thresholds, which were previously associated with adverse ischemic outcomes in patients with cardiovascular disease [7–10], to define HRPR in response to ADP (HRPR ADP) and HRPR in response to AA (HRPR AA) for LTA, the VerifyNow assays and MEA. The corresponding cut-off values for HRPR ADP were a maximal aggregation >67% for LTA ADP, PRU >235 for the VerifyNow P2Y_12_ assay, and AU ≥47 for MEA ADP [7]. Thereby, HRPR ADP was seen in 15.3%, 33.7%, and 38.3% of the patients by LTA ADP, the VerifyNow P2Y12 assay, and MEA ADP, respectively. The platelet surface expressions of P-selectin and activated GPIIb/IIIa in response to ADP were significantly higher in patients with HRPR ADP by all test systems (all p<0.001).

The cut-off values for HRPR AA were a maximal aggregation ≥20% for LTA AA [10], ARU ≥550 for the VerifyNow aspirin assay [8], and AU ≥21 for MEA AA [9]. Thereby, HRPR AA was seen in 10.5%, 12.7%, and 29% of the patients by LTA AA, the VerifyNow aspirin assay, and MEA AA, respectively. The platelet surface expression of P-selectin in response to AA was significantly higher in patients with HRPR by LTA AA and MEA AA (both p<0.02). In contrast, P-selectin expression in response to AA was similar in patients without and with HRPR by the VerifyNow aspirin assay (p = 0.5), and platelet surface activated GPIIb/IIIa in response to AA did not differ significantly between patients without and with HRPR AA by all test systems (all p>0.1).

Finally, agonists´-induced platelet surface expression of P-selectin and activated GPIIb/IIIa in the fourth quartile was defined as high P-selectin and high GPIIb/IIIa, respectively. The resulting cut-off values were a fold change ≥5.7 for high P-selectin and a fold change >5.2 for high GPIIb/IIIa in response to ADP. With use of these thresholds, high P-selectin in response to ADP was seen in 71.7%, 47%, and 36.5% of the patients with HRPR by LTA ADP, the VerifyNow P2Y_12_ assay and MEA ADP, respectively. High GPIIb/IIIa in response to ADP was seen in 63%, 54.5%, and 41.4% of the patients with HRPR by LTA ADP, the VerifyNow P2Y_12_ assay and MEA ADP, respectively. Thereby, high P-selectin and high activated GPIIb/IIIa in response to ADP were significantly more frequent in patients with HRPR ADP by all aggregation tests compared to patients without HRPR ADP (all p≤0.001).

The cut-off values for high P-selectin and high GPIIb/IIIa in response to AA were a fold change >2.3 and a fold change >2.6, respectively. With use of these thresholds, high P-selectin in response to AA was seen in 42.4%, 23.1%, and 39.3% of the patients with HRPR by LTA AA, the VerifyNow aspirin assay and MEA AA, respectively. High GPIIb/IIIa in response to AA was seen in 30.3%, 25.6%, and 30.3% of the patients with HRPR by LTA AA, the VerifyNow aspirin assay and MEA AA, respectively. Thereby, high P-selectin was significantly more frequent in patients with HRPR by LTA AA and MEA AA (both p<0.02). In contrast, high AA-induced P-selectin did not differ between patients without and with HRPR by the VerifyNow aspirin assay, and high AA-induced GPIIb/IIIa did not differ significantly between patients without and with HRPR by all aggregation tests (all p>0.1).

## Discussion

Our study is the first to compare platelet aggregation by three different test systems and platelet activation by flow cytometry for the assessment of on-treatment platelet reactivity to ADP and AA in patients on dual antiplatelet therapy. While ADP-induced platelet reactivity by aggregometry was at least in part reflected by flow cytometry, AA-induced platelet reactivity by all aggregation tests correlated poorly with AA-induced P-selectin expression and activated GPIIb/IIIa, respectively. Moreover, platelet surface expressions of P-selectin and activated GPIIb/IIIa in response to ADP were significantly more pronounced in patients with HRPR ADP by all aggregation tests, and high ADP-induced P-selectin and activated GPIIb/IIIa were significantly more frequent in patients with HRPR ADP. In contrast, higher levels of AA-induced P-selectin expression were only seen in patients with HRPR by LTA AA and MEA AA, and the expression of activated GPIIb/IIIa in response to AA did not differ between patients without and with HRPR AA by all test systems. Further, the associations of high P-selectin and activated GPIIb/IIIa in response to AA with HRPR AA were less pronounced than those between ADP-induced activation and aggregation assays.

We selected the three most frequently used platelet aggregation tests for the comparisons with flow cytometry. LTA is considered the historical gold standard for the assessment of on-treatment platelet reactivity, and its results were associated with clinical outcomes in numerous studies [10, 13]. The major drawbacks of LTA are its lack of standardization and the two centrifugation steps required to generate PRP and PPP, respectively. Both affect the comparability of results by LTA between different laboratories, and the latter makes the method quite time-consuming. Moreover, by using plasma instead of whole blood, LTA creates an artificial milieu, which excludes the interaction of platelets with other blood cells. In contrast to LTA, the VerifyNow assays and MEA are fast and well-standardized whole blood assays [5]. While the VerifyNow assays are based on the same principle like LTA, i.e. the increase of light transmittance through a sample after the initiation of platelet aggregation, MEA measures the increase of impedance between two electrodes when platelets are activated and start to adhere to them. The results of both methods have been repeatedly linked to the occurrence of adverse ischemic events in patients with cardiovascular disease [7–9, 14, 15].

The surface expressions of P-selectin and activated GPIIb/IIIa are both considered sensitive markers of platelet activation. Platelet surface P-selectin interacts with P-selectin glycoprotein ligand-1 on leukocytes, and thereby plays a critical role in tethering these cells to activated platelets [16]. The resulting monocyte-platelet aggregates were shown to be elevated in myocardial infarction and in stable coronary artery disease [17, 18]. Activated GPIIb/IIIa mediates the interaction of platelets with coagulation factors and other platelets [19]. Both P-selectin and activated GPIIb/IIIa were recently associated with adverse ischemic outcomes in patients undergoing angioplasty and stenting for peripheral arterial disease [11]. Of note, all used test systems, i.e. aggregometry and flow cytometry, are *in vitro* assays and must therefore be considered as non-physiological.

In the last years, others and we compared different platelet aggregation tests for the assessment of ADP- and AA-induced platelet reactivity [20–25]. In line with the current study, the best correlations among those were observed between LTA ADP and the VerifyNow P2Y_12_ assay [20, 22, 24, 25]. This may be due to the above-mentioned fact that both methods are based on the same principle. In the current study, the correlations between LTA ADP and MEA ADP, and between the VerifyNow P2Y_12_ assay and MEA ADP were weaker but statistically still highly significant. In contrast, AA-induced platelet reactivity correlated only weakly between LTA AA and the VerifyNow aspirin assay in the current study. Further, LTA AA and MEA AA as well as the VerifyNow aspirin assay and MEA AA did not correlate. This is in line with previous observations revealing poor correlations between different platelet function tests assessing the response to aspirin therapy [21, 23], and suggests that the various methods capture different aspects of AA-induced platelet reactivity.

In our study, the best correlations of aggregometry were seen with activated GPIIb/IIIa. This may be attributable to the fact that activated GPIIb/IIIa is directly involved in platelet aggregation, while P-selectin mediates mainly the interactions between platelets and leukocytes. Overall, the correlations of ADP-induced platelet reactivity between aggregometry and flow cytometry were similar to those among the three platelet aggregation tests. The best correlation was found between the VerifyNow P2Y_12_ assay and ADP-induced activated GPIIb/IIIa. The observed correlation coefficient was similar to a previous study, in which Godino et al. assessed platelet activation by flow cytometry and on-treatment platelet aggregation by the VerifyNow P2Y_12_ assay [26]. In their study, they defined low response to clopidogrel therapy based on flow cytometry measurements in 30 healthy controls and 52 patients with stable angina. In a second step, they performed receiver-operating characteristic curve analyses to determine cut-off values of ADP-induced platelet reactivity by the VerifyNow P2Y_12_ assay, which were able to identify patients with low response to clopidogrel. While they focused on reproducing flow cytometric results on ADP-induced platelet activation with one point-of-care assay, we compared the three most widely used platelet aggregation tests with agonists’-induced platelet surface expression of P-selectin and activated GPIIb/IIIa. Moreover, in order to address the response to antiplatelet therapy with both, clopidogrel and aspirin, we measured not only ADP- but also AA-induced platelet aggregation and activation. Besides the significant correlations between ADP-induced platelet aggregation and activation, we found significantly higher levels of platelet surface P-selectin and activated GPIIb/IIIa in response to ADP in patients with HRPR ADP by all aggregation tests. Finally, high ADP-induced P-selectin and activated GPIIb/IIIa were significantly more frequent in patients with HRPR ADP compared to patients without HRPR ADP by all aggregation tests.

AA-induced P-selectin expression correlated significantly but rather poorly with the three aggregation tests, while AA-induced activated GPIIb/IIIa correlated only with LTA AA. Furthermore, the expression of activated GPIIb/IIIa in response to AA did not differ between patients without and with HRPR AA by all test systems, and the associations of high P-selectin and activated GPIIb/IIIa with HRPR were less pronounced when AA was used as an agonist. Consequently, in particular the methods for the assessment of the response to aspirin are not interchangeable, and the decision for one of these test systems should be based on clinical data only.

We used lithium heparin instead of hirudin as anticoagulant for MEA. Recently, Kalb et al. compared platelet aggregability by MEA in whole blood stored in citrate, heparin and direct thrombin inhibitors [27]. They found no significant differences between samples containing direct thrombin inhibitors and samples containing heparin at baseline. In contrast, aggregation by MEA was significantly impaired in citrate-anticoagulated blood. During storage the response to AA was maintained by direct thrombin inhibitors and heparin, whereas ADP-induced aggregation varied considerably over time in all *ex vivo* anticoagulants tested. However, ADP-induced platelet aggregation at 2 hours did not differ significantly between hirudin- and heparin-anticoagulated whole blood. The authors concluded that unfractionated heparin is also suitable as anticoagulant for MEA.

A limitation of our study is the lack of clinical outcome data. Consequently, we report correlations and agreement between different *in vitro* tests, and the clinical relevance of our findings remains unclear.

In conclusion, ADP-induced platelet reactivity by aggregometry translates partly into flow cytometry. In contrast, AA-induced platelet reactivity correlates poorly between different platelet aggregation tests, and between aggregometry and flow cytometry. Overall, both approaches capture different aspects of platelet function and are therefore not interchangeable in the assessment of agonists´-induced platelet reactivity. Clinical outcome data are needed to determine which test systems and settings are associated with different *in vivo* consequences [28].
